# Digital vs. conventional workflow for one-abutment one-time immediate restoration in the esthetic zone: a randomized controlled trial

**DOI:** 10.1186/s40729-022-00406-6

**Published:** 2022-02-07

**Authors:** Brieuc Hanozin, Lou Li Manni, Geoffrey Lecloux, Miljana Bacevic, France Lambert

**Affiliations:** 1 Department of Periodontology, Oral and Implant Surgery, University Hospital of Liège, Domaine du Sart Tilman Bat B-35, 4000 Liège, Belgium; 2 Dental Biomaterials Research Unit (d-BRU), University of Liège, 4000 Liège, Belgium

**Keywords:** s-CAIS surgery, Fully digital workflow, One-abutment one-time, White esthetic score (WES), Patient-reported outcome measures (PROMs), Custom-made abutment

## Abstract

**Objectives:**

To compare short-term outcomes after immediate restoration of a single implant in the esthetic zone with one-abutment one-time technique comparing a conventional (control) vs. a fully digital workflow (test).

**Materials and methods:**

Eighteen subjects were randomly assigned to the two groups, and a digital implant planning was performed for all. In the test group, a custom-made zirconia abutment and a CAD–CAM provisional crown were prepared prior to surgery; implants were placed using a s-CAIS guide allowing immediate restoration after surgery. In the control group, the implant was placed free-handed using a conventional surgical guide, and a custom-made zirconia abutment to support a stratified provisional crown was placed 10 days thereafter, based on a conventional impression. Implant accuracy (relative to the planning), the provisional restoration outcomes, as well as PROMs were assessed.

**Results:**

The implant positioning showed higher accuracy with the s-CAIS surgical guide compared to free-handed surgery (angular deviation (AD): 2.41 ± 1.27° vs. 6.26 ± 3.98°, *p* < 0.014; entry point deviation (CGD): 0.65 ± 0.37 mm vs. 1.27 ± 0.83 mm, *p* < 0.059; apical deviation (GAD): 1.36 ± 0.53 mm vs. 2.42 ± 1.02 mm, *p* < 0.014). The occlusion and interproximal contacts showed similar results for the two workflows (*p* = 0.7 and *p* = 0.69, respectively). The PROMs results were similar in both groups except for impression taking with intra-oral scanning preferred over conventional impressions (*p* = 0.014).

**Conclusions:**

Both workflows allowed implant placement and immediate/early restoration and displayed similar clinical and esthetic outcomes. The fully digital workflow was associated with a more accurate implant position relative to planning.

**Clinical relevance:**

Our results show that both conventional and digital workflow are predictive and provide similar clinical outcomes, with extra precision provided by digitalisation.

## Introduction

Prosthetically driven implant dentistry is the optimal way to treat patients with dental implants [1]. It requires detailed pretreatment planning to ensure a correct three-dimensional implant position, relative to the planned prosthetic restoration [2]. Correct implant positioning has obvious advantages, such as favorable esthetic and prosthetic outcomes, as well as the long-term stability of peri-implant hard and soft tissues. In fact, the soft tissues play the role of a barrier, which prevents bacterial progression from the oral cavity to the implant surface [3]. For instance, on bone level implants, repeated unscrewing of the abutment was found to lead to a rupture of the soft tissue integration and induced apicalisation of biological width and pocket formation that may compromise the peri-implant soft tissues’ integrity [4–6]. An often described concept to avoid repeated abutment removal is the “one-abutment one-time” approach, consisting of the final abutment placement at the time of surgery with a prefabricated abutment [7–10]. It was described in the literature that the position of finishing line and the abutment emergence profile are of extreme importance to avoid cement fusion [11–13]. The use of an individualized abutment with an optimal finishing line and an ideal emergence profile would help to prevent such complications. Up to now, an impression just after the surgery and a period of about a week has been necessary to design and manufacture a custom-made abutment, optimal for one-abutment one-time procedures in the esthetic zone.

Recently, an advanced digital technology for pre‐operative implant planning called static computer‐assisted implant surgery (s-CAIS) has allowed simultaneous visualization of three‐dimensional (3D) bone morphology, soft tissues of the alveolar ridge, and teeth [14–16]. This technique uses computer technology for virtual implant position planning prior to surgery according to the bone quality and quantity, the location of important anatomical structures, soft tissues and teeth, and the functional and esthetic demands of future prostheses [17]. During the surgical intervention, the planned implant position is transferred to the surgical site by a 3D‐printed surgical guide [18]. This technique can potentially prevent complications, such as nerve damage, sinus perforation, fenestration or adjacent tooth damage [15, 19]. However, deviations from the planned position were reported due to the sum of errors that may occur from imaging to data translation into the s-CAIS guide or due to an improper positioning of the guide during surgery [20, 21].

Although s-CAIS is gaining popularity in the practice of implant dentistry, concerns about its accuracy have not been fully addressed [22, 23]. The accuracy of implant positions placed by s-CAIS has been investigated using preoperative and postoperative cone-beam computed tomography (CBCT) scans in several recent studies [15, 23–30]. These studies have reported high accuracy and more precise implant positions for future restoration with the use of s-CAIS, but the results encompass a wide range of outcomes, as different settings have been utilized in most studies and most are only based on in vitro models. Taken together, we believe that there is a further need for clinical data from randomized studies to support our understanding of the accuracy of s-CAIS and the important factors for decision‐making in a clinical setting.

Moreover, the newly available digital tools allow the fabrication of final custom-made abutment and computer-aided design and computer-aided manufacturing (CAD–CAM) provisional crown prior to the surgery, based on a surgical and prosthodontic planning. Consequently, a possibility to prepare individualized prosthodontic components prior to surgery allows immediate implant provisionalization using a one-abutment one-time approach and a fully digital workflow. However, even though case series using a fully digital workflow were published [31], the current literature still lacks randomized clinical trials comparing a conventional implant protocol with a digital workflow to further validate such novel protocols.

The overall objectives of the present randomized controlled trial were to assess the accuracy and reliability, the potential prosthetic benefits, as well as the patient centered outcomes of a fully digital workflow vs. a conventional approach for placement and immediate loading of a single implant in the esthetic zone using an individualized one-abutment one-time protocol. The primary aim was to compare the accuracy of the implant position in the esthetic area—free-handed vs. fully guided. The hypothesis was that the digital workflow using guided surgery was more accurate than the conventional procedure employing free-handed surgery. The secondary objectives were to evaluate the short-term loading outcomes, the fit and potential adjustments of the provisional crowns, as well as esthetics and patient satisfaction in the two groups.

## Materials and methods

### Study design

**Fig. 1:**
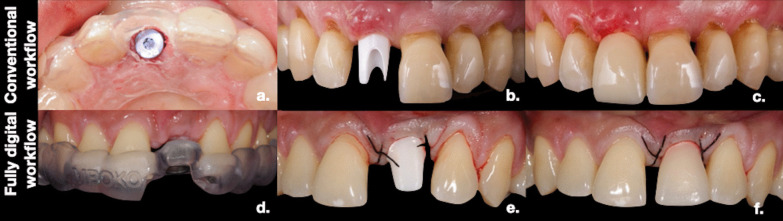
Clinical steps of the conventional and fully digital workflows: Conventional group: **a** surgical guide in the control group; **b** placement of the custom-made zirconia abutment at 10 day post-surgery; **c** provisional crown made of stratified PMMA. Fully digital group: **d** s-CAIS guide; **e** placement of the custom-made abutment right after surgery; **f** provisional CAD–CAM crown

**Fig. 2:**
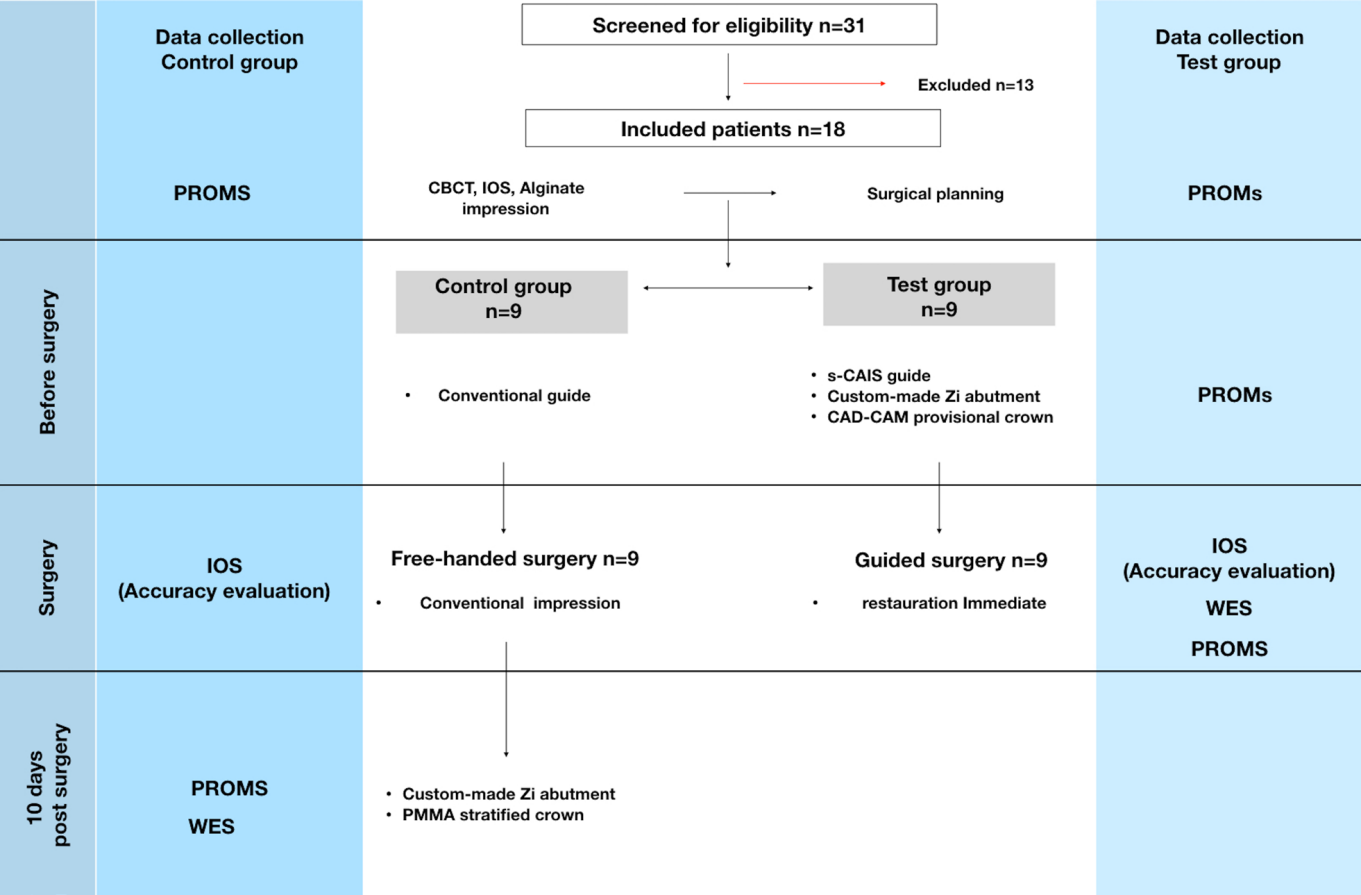
Study design: Consort flow chart. *CBCT* cone beam computed tomograpy, *WES* white esthetic score, *PROMs* patient-reported outcome measures, *IOS* intra oral scan

**Fig. 3:**
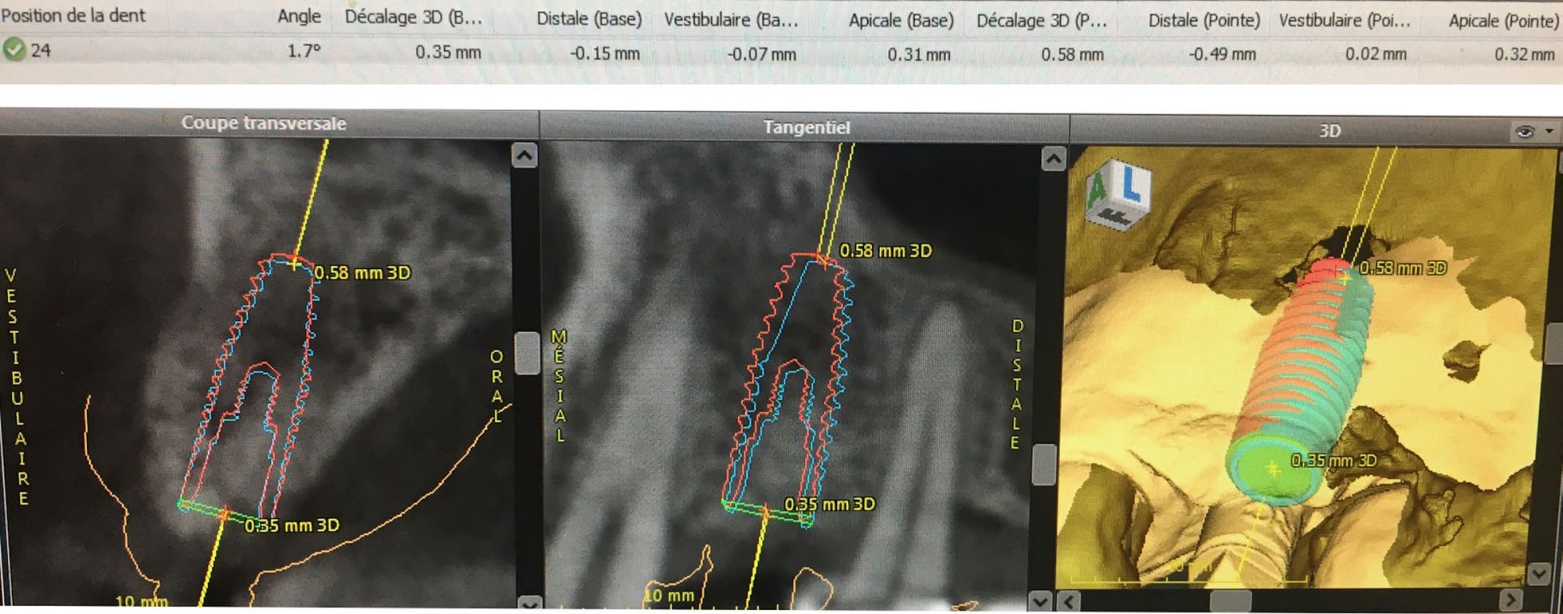
Deviation measurement from treatment evaluation mode in coDiagnostiX^®^ software version 9.7 (Dental wings Inc.). Implant illustrate (red line): final implant position, (blue line): planned implant position, Angle (°): angle deviation in degrees, Base (mm): deviation at implant shoulder in mm, Tip (mm): deviation at implant apex in mm, 3D offset: deviation in three‐dimensional directions, Distal: deviation to mesiodistal direction, + : deviated to distal direction, − : deviated to mesial direction, Vestibular: deviation at buccolingual direction, + : deviated to lingual direction, − : deviated to buccal direction, Apical: deviation at apicocoronal direction. + : deviated to apical direction. − : deviated to coronal direction

The present study was designed as a randomized controlled trial comparing immediate restoration of single implants in the esthetic zone with a one-abutment one-time technique and using a conventional (control group) vs. full digital workflow (test group) (Figs. 1, 2). The primary endpoint of the study was to assess the accuracy of the implant position, which was measured using the global–apical deviation (GAD) between the post-surgical implant position and the digital planning in each group (Fig. 3).

The sample size calculation was construed to detect a difference of 1 mm at the apical implant position with a standard deviation of 0.5 mm for the full guided surgery as adopted by Van Assche [27] and 0.8 mm for the control group at a power of 80% with a significance level (alpha) of 0.05 using the Satterthwaite *t* test. According to this calculation, 18 patients were included in the study (9 per group).

Patients were treated in the Department of Periodontology, Oral and Implant Surgery of the University of Liège and recruited from January 2018 through June 2019. Two senior periodontists were involved in the surgical procedures and in the immediate restoration procedures, and the short-term outcomes were collected over a period of 10 days until suture removal. Possible patient dropouts and withdrawals, as well as adverse events, were carefully monitored during the investigation period. The study protocol was approved by the Ethical Committee of the University Hospital of Liège, Belgium (file number: B707201731117). The study was registered on clinicaltrial.gov (file number: NCT04139512) and was performed according to the CONSORT statement for transparent reporting of randomized clinical trials (http://www.consort-statement.org/).

### Inclusion/exclusion criteria

Each of the patients met the following inclusion criteria: patient over 18 years in good general health (ASA I or II), presenting a single missing tooth or 2 non-adjacent missing teeth in the esthetic area of the upper jaw (from 15 to 25, according to Tjan et al. [32]) or requiring an extraction and immediate implant, healthy periodontal condition and full mouth plaque score (FMPS) lower or equal to 25%, at least 10 mm of bone in the vertical dimension, at least 7 mm of bone in the bucco-palatal dimension, no need for simultaneous bone augmentation procedure (however, a bone augmentation procedure could have been realized ≥ 4 months prior to the digital planning of surgery). Every subject voluntarily signed the informed consent form before any study related action.

Exclusion criteria were as follows: current pregnancy or breastfeeding women, alcoholism or chronical drug abuse, immunocompromised patients, uncontrolled diabetes, smokers > 10 cigarettes per day, use of bisphosphonates intravenously or more than 3 years of oral use, autoimmune disease requiring medical treatment, medical conditions requiring prolonged use of steroids, infection (local or systemic). Each infection was evaluated prior to study procedure for suitability. Patients with gingivitis or local infection underwent a treatment prior to entrance to the study. In case of a systemic infection, the evaluation was based on medical anamnesis and, if necessary, the patient was referred to perform other relevant medical tests. Moreover, untreated local inflammation, mucosal disease or oral lesions, history of local irradiation therapy in the head–neck area, persistent intraoral infection, lack of motivation for normal home care were considered as local exclusion criteria.

### Procedure

For both groups, a digital impression (TRIOS^®^, 3Shape, Denmark) and conventional alginate impressions were made. The Digital Imaging and Communications in Medicine (DICOM) file from the CBCT examination and the Standard Tessellation Language (STL) file from the surface scan were imported and merged in coDiagnostiX^®^ software version 9.7 (Dental Wings Inc., Montreal, Canada). The virtual implant planning for all patients was performed by one qualified dentist with significant experience in implant dentistry while considering the prosthetic target. Implant dimension and position were chosen and determined according to the available bone volume and 3D implant positioning standard [2]. The lab technician was informed about the randomization by a third person.

#### Full digital workflow: test group

In the test group, a digital tooth setup was performed in the CARES software. The setup was fused with the initial implant planification and modification of the implant position was performed to provide the best situation regarding the future tooth position. Once the final planning was validated, a fully guided drill guide (Dreve, Dentamid GmbH, Unna, Germany) was ordered. A guided Bone Level Tapered implant (BLT; 3.3 mm or 4.1 mm diameter, RC, Roxolid^®^, SLActive^®^, Institut Straumann AG, Basel, Switzerland) was used. According to the planned implant position, in the CARES software, the lab technician digitally designed the custom-made zirconia abutment and the CAD–CAM polymethyl methacrylate (PMMA) provisional crown and ordered those components (etkon^®^ iDent/Medentika^®^) for the day of the surgery. The design of the final Zirconia CARES^®^ X-Stream abutment (Institut Straumann AG, Basel, Switzerland) was made according to the digital setup of the lab and planned as final for a cemented restoration. The finishing lines were determined to be 0.5 mm submucosally to the planned teeth setup, taking into consideration the shrinkage of the soft tissues after implant surgery, and the transgingival profile were designed with a concave and umbrella effect contour. A silicon verification key on the buccal side of the surgical guide was provided by the laboratory to check the implant position during the surgery. The patients received anti-inflammatories (ibuprofen 600 mg) the day before surgery and for 5 days after surgery. Additional analgesics (paracetamol) were prescribed according to the patient’s needs. Prior to the patient installation in the O.R., the surgical templates (free-handed or full-guided) were tried in mouth and adjusted to avoid mucosal contact. After local anesthesia, a full thickness but minimally invasive flap was elevated above the treatment site to allow access to the site. In case of extraction and immediate implant placement, a sufficient apical anchorage was necessary. Therefore, the buccal gap was filled with bovine hydroxyapatite (cerabone^®^, botiss biomaterials GmbH, Zossen, Germany) and combined with a connective tissue graft placed transmucosally on the buccal part of the healing abutment (control)/final abutment (test) group. Implant insertion torque was measured using a torque wrench and was recorded in N/cm. Immediate loading was performed only if a primary stability of 35 N/cm was achieved.

After implant placement, a digital impression of implant’s position was performed using the TRIOS^®^ intraoral scanner (3Shape, Denmark) and implant-specific scan bodies. Then, the custom-made abutment and provisional crown were tried to check the vertical, bucco-lingual and mesio-distal position. After the fit of the components was verified, the provisional crown was immediately loaded with the final custom-made zirconia abutment. If possible, the crowns were cemented outside the mouth with a resin-based temporary cement (Seal Temp S, Elsodent, France) and if the screw channel access did not allow it, the cementation was performed before suturing to allow a visual control of cement removal. Finally, the crown was adjusted to be out of centric and lateral occlusion. A chlorhexidine spray (0.12%) was prescribed twice daily on the surgical sites for 7 days in addition to ibuprofen, 600 mg TID, prescribed for 4–5 days. Patients were advised to avoid tooth brushing at the implant site for 7 days. The sutures were removed 10 days after surgery.

#### Conventional workflow: control group

In the control group, an orthocryl free-hand surgical guides based on a conventional wax up were ordered and a custom-made open impression tray was made by the laboratory. During the surgery, the consecutive drills of the implantation procedure were carried through the free-hand guide to position the implant according to the prosthodontic planning. A guided Bone Level Tapered implant (BLT; 3.3 mm or 4.1 mm diameter, RC, Roxolid^®^, SLActive^®^, Institut Straumann AG, Basel, Switzerland) was used. Directly after the implant surgery, a physical impression was made with a custom-made open tray and open tray transfer with Aquasil^®^ Ultra + Medium (Dentsply Sirona, York, USA). Immediately after the disinsertion of the impression, the dentist placed the implant replica in the impression and the impression was sent to the lab. Based on the model and the replica of the implant, the lab designed the final Zirconia CARES^®^ X-Stream abutment (Institut Straumann AG, Basel, Switzerland) according to the wax-up. The finishing lines were determined to be 0.5 mm submucosally to the planned wax-up and the transgingival profiles were designed with a concave and umbrella effect contour. Afterwards, a PMMA stratified provisional crown was produced. The custom-made abutment, the provisional crown, and the verification key were then sent to the clinicians. Ten days after the surgery, the early loading of a control group implant was performed according to the protocol described for the test group on the day of the surgery.

### Assessment of the surgical accuracy

**Fig. 4:**
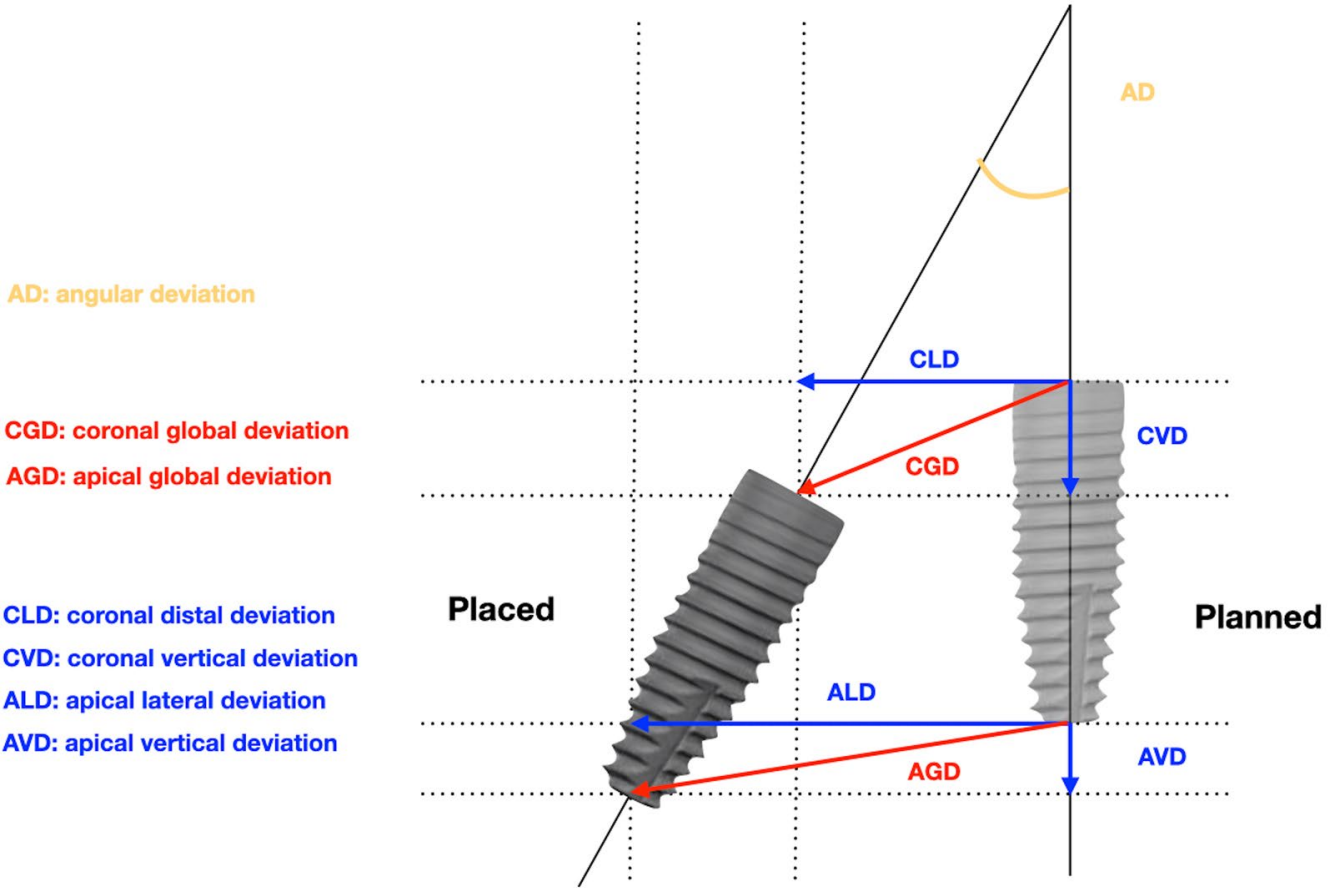
Parameters of deviation between the planned implant (right) and actual implant (left) position. *AD* angular deviation, *CGD* coronal global deviation, *GAD* global apical deviation, *CDD* coronal distal deviation, *ADD* apical distal deviation, *CAD* coronal apical deviation, *AAD* apical apical deviation, *CVD* coronal vestibular deviation, *AVD* apical vestibular deviation

For both groups, the passive insertion (yes or no) and possible adjustments (yes or no) of the surgical guides were recorded. Right after the implant placement, a digital impression (TRIOS^®^, 3Shape, Denmark) was made using digital intraoral scans with respective scan bodies. The STL files were superimposed with the pre‐operative CBCT images using an automated surface best fit matching with the iterative closest point algorithm in the treatment evaluation mode, coDiagnostiX^®^ software version 9.7 (Dental Wings Inc.). The mean deviations at the implant shoulder and apex between the planned and actual implant positions were measured in millimeters (mm), as well as the divergence of the implant axis in degrees. All measurements were performed by a single dentist, who conducted the virtual implant planning in all cases (Fig. 3). The following nine parameters were analyzed (Fig. 4):

- Angular deviation (AD)
- Coronal global deviation (CGD)
- Global apical deviation (GAD)
- Coronal distal deviation (CDD)
- Apical distal deviation (ADD)
- Coronal apical deviation (CAD)
- Apical apical deviation (AAD)
- Coronal vestibular deviation (CVD)
- Apical vestibular deviation (AVD)

### Assessment of the provisional restoration outcomes

Loading outcomes were recorded on the day of crown placement and were based on the clinical fit of the provisional restoration. When the provisional restoration crown could not be placed because of severe inaccuracy, it was considered as a workflow failure. The measurements were based on the adaptation of the provisional crown. Interproximal contacts were evaluated according to the criteria described by Syrek [33]. A dental floss (Johnson & Johnson Reach^®^, New Brunswick, NJ, USA) was used to check the interproximal contact. A score from 1 to 4 was given to the fitting criteria as follows (the higher the score, the better the adaptation):

1. Clinically poor: No contact point, or papilla damage, or crown cannot be seated.
2. Clinically unsatisfactory: Contact too tight, dental floss cannot be inserted, or contact too open with food impaction likely to occur.
3. Clinically good: Contact slightly tight, but dental floss can still be inserted.
4. Clinically excellent: Normal contact point; dental floss can be easily inserted.

As for the assessment of the temporary crown occlusion in case of immediate implant restoration, no validated method is described in the literature. Therefore, the following criteria were defined:

1. Clinically poor: Overcontact on the provisional crown (supraocclusion).
2. Clinically good: Occlusal contact points on the crown and adjacent teeth present but unequal in strength; no supra- or infraocclusion.
3. Clinically optimal: The provisional crown is out of centric and eccentric occlusion (infraocclusion).

Static and dynamic occlusion was assessed with shimstock occlusion foil (Bausch Arti-Check^®^, Köln, Germany).

If applicable, corrections of the implant crowns were made using a diamond bur and a silicone polisher.

In addition, to evaluate the esthetic outcomes of the provisional crown, the White Esthetic Score (WES) was reported. The highest WES score was 10, which represented a close match with the clinical single-tooth crown present at the contralateral natural tooth or neighboring teeth. The WES was evaluated by a blinded prosthodontist, based on photography. In addition, patients’ opinions on the esthetic results were also collected using an esthetic visual analogue scale (VAS, from 0 to 10, 10 being the most esthetic and natural-looking). A possible correlation between the VAS and WES scores was explored.

To evaluate the esthetic outcome of soft tissue around restorations, the Pink Esthetic Score (PES) was used. The PES was evaluated by a blinded prosthodontist, based on photography. Seven standard variables were evaluated: mesial papilla, distal papilla, soft tissue level, soft tissue contour, alveolar process deficiency, soft tissue colour, and soft tissue texture. The 0–1–2 scoring system was used, 0 being the lowest and 2 being the highest value; therefore, the maximum score was 14.

### Patient-reported outcome measures (PROMs)

PROMs were obtained using a VAS form immediately after the immediate restoration of the implant. For each group, the following five questions were asked:

1. Do you feel your provisional tooth as a natural tooth? (0 = not at all to 10 = absolutely).
2. Do you find your provisional tooth looks like a natural tooth? (0 = not at all to 10 = absolutely).
3. How much discomfort did you feel during the physical impression? (0 = little or 10 = a lot) for the control group.
  How much discomfort did you feel during the optical impression? (0 = little to 10 = a lot) for the test group.
4. Would you be willing to undergo this treatment again? (0 = not at all to 10 = absolutely).
5. Are you satisfied with the esthetic outcomes of your provisional tooth? (0 = not at all to 10 = absolutely).

### Statistics

The results are presented as means, standard deviation (SD), median, quartiles (Q1–Q3), and extrema for continuous variables and as frequency tables for quantitative variables. Continuous variables were compared between the groups by Student’s *T* test or Kruskal–Wallis test and qualitative variables by the Chi-square test or Fisher’s exact test. To compare the gaps between the groups and sites, the GLMM model was used. The association between continuous variables was measured by the Pearson correlation coefficient. Results were considered significant at 5% uncertainty level (*p* < 0.05). Calculations were done using SAS version 9.4 and the figures were made using R version 3.5.

## Results

### Demographics

A total of 31 patients were screened for potential inclusion in the present study, and 18 met the inclusion criteria. The main causes of exclusion were: tobacco habits (8 patients), bruxism (4 patients), and less than 10 mm in vertical bone dimension (1 patient). A total of 18 patients, 9 in each group, were enrolled in this study and received 18 BLT implants varying from 10 to 14 mm in length in the esthetic zone (maxillary incisors or premolars) (Table 1). The mean age was 57 years in the test group and 47 years in the control group. Both groups were homogenous regarding age (*p* = 0.13), gender (*p* = 0.29), implant diameter (*p* = 0.64), implant length (*p* = 0.21), implant position (*p* = 1.00), and the type of implantation site (*p* = 0.66).

**Table 1:** Patient and implant related characteristics

	Test (*N* = 9)	Control (*N* = 9)	*p*-value
Patient			
Age	47.67	57.11	0.13
Gender			
F	5	8	0.29
M	4	1	
Implant			
Diameter (mm)			
3.3	3	5	0.64
4.1	6	4	
Length (mm)			
10	0	3	0.21
12	8	5	
14	1	1	
Implant position			
Central incisor	3	3	1.0
Lateral incisor	3	2	
Premolar	3	4	
Site			
Natural healing	1 (22.2)	3 (11.1)	0.66
Regenerated	6 (66.7)	5 (55.6)	
Extraction immediate implantation	1 (11.1)	3 (33.3)	
Possibility of guided insertion			
Yes	8 (88.9)	9 (100.0)	1.00
No	1 (11.1)	0 (0.00)	

### Surgical outcomes

**Fig. 5:**
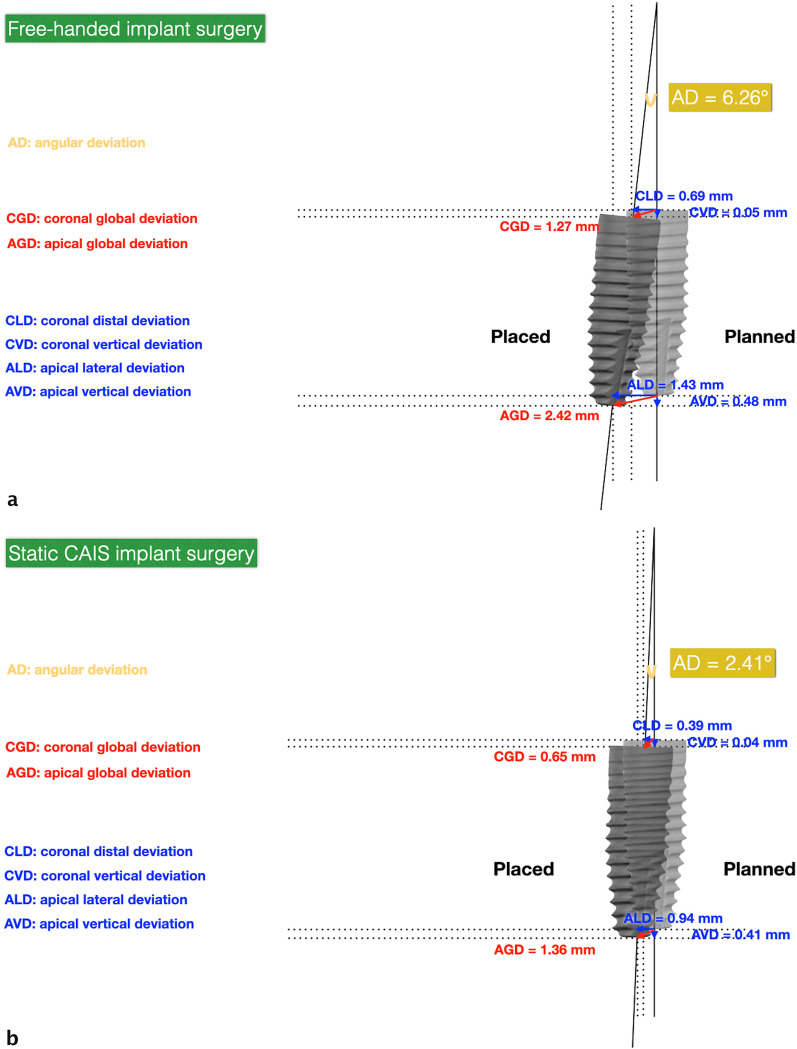
Parameters of deviation between the planned implant (right) and actual implant (left) position in the free-handed (**a**) and static CAIS (**b**) implant surgery. Angular deviation (AD) is presented to scale, while the other deviations are not to scale and only serve for better visualization. The presented values are mean values taken from Table 2

In the control group, the insertion of the conventional guide was possible for all patients and no modifications were needed. In the test group, one surgical guide could not be immediately inserted and positioned and needed some minor adjustments. The higher deviation at the entry point (1.13 mm) in the test group was found for the guided surgery, which needed modification. In addition, for one case in the test group, a vertical deviation of 2 mm in depth was observed clinically by the surgeon, who decided to place a 12 mm implant instead of a 10 mm one as intended according to the digital planning. The surgical accuracy parameters between the final implant position and the digital planning for both groups are displayed in Table 2. The global apical deviation (GAD) was significantly higher in the control group compared to the test group (*p* = 0.014). In addition, significant differences were observed for the AD (*p* = 0.014) and the AVD (*p* = 0.038) in favor of the fully guided surgery (Fig. 5a, b). Data analysis did not demonstrate statistically significant differences between the two groups regarding CGD, CDD, CVD, CAD, ADD and AAD values.

**Table 2:** Deviation of implant position in static CAIS and free-handed implant surgery groups

*N* = 18	Mean	SD	Min	Max	*p*-value
AD (angulation deviation)					
Control	6.260	3.977	0.900	11.40	0.014
Test	2.410	1.267	0.710	4.000	
CGD (entry point deviation)					
Control	1.270	0.831	0.470	2.310	0.059
Test	0.650	0.373	0.150	1.130	
CDD					
Control	0.690	0.612	0.030	1.940	0.200
Test	0.390	0.238	0.020	0.610	
CVD					
Control	0.720	0.586	0.060	2.140	0.230
Test	0.440	0.365	0.070	1.020	
CAD					
Control	0.050	0.373	0.090	1.200	0.480
Test	0.039	0.290	0.100	0.950	
GAD (apical deviation)					
Control	2.420	1.017	1.150	4.620	0.014
Test	1.360	0.529	0.580	2.310	
ADD					
Control	1.430	0.733	0.470	2.570	0.130
Test	0.940	0.545	0.240	1.850	
AVD					
Control	1.740	1.038	0.060	3.810	0.038
Test	0.810	0.654	0.020	2.250	
AAD					
Control	0.480	0.369	0.030	1.230	0.660
Test	0.410	0.281	0.080	0.920	

*AD* angular deviation, *CGD* coronal global deviation, *CDD* coronal distal deviation, *CVD* coronal vestibular deviation, *CAD* coronal apical deviation, *GAD* global apical deviation, *ADD* apical distal deviation, *AVD* apical vestibular deviation, *AAD* apical apical deviation; AD is expressed in degrees; all the other deviations are expressed in mm. *SD* standard deviation, *Min* minimum, *Max* maximum

### Provisional restoration outcomes

A primary stability of 35 Ncm was achieved for all implants; all custom-made zirconia abutments were torqued to 15 Ncm and all provisional restorations could be placed—immediately in the test group and after 10 days in the control group.

The occlusal and interproximal fit of the provisional restoration did not show any statistically significant differences between the two groups (occlusion: *p* = 0.70 and interproximal contact: *p* = 0.69). However, occlusal adjustments were necessary in about half of the cases in both groups to be out of centric and eccentric occlusion, and interproximal contact adjustments were more often needed in the test group (55.6%).

The WES was comparable in the two groups (*p* = 0.45); however, the data revealed a trend to have a higher score in the surface texture with the conventional stratified provisional crown compared to the CAD–CAM one (*p* = 0.05). The PES score tended to be higher in the test group (*p* = 0.057). In terms of soft tissue colour and soft tissue texture, the values of “2” were recorded for all patients in the test group, while the proportion of score “2” was 44.4% in the control group (*p* = 0.029, respectively). The details of the results are shown in Table 3.

**Table 3:** Loading procedure outcomes, occlusion and interproximal contacts (%), WES, and PES results

	Test (%)	Control (%)	*p*-value
Successful loading procedure			
Yes	9 (100.0)	9 (100.0)	NA
No	0 (0.0)	0 (0.0)	
Occlusion			
1: Supraoclusion	1 (11.1)	3 (33.3)	0.7
2: Limited contact	4 (44.4)	3 (33.3)	
3: Infraoclusion	4 (44.4)	3 (33.3)	
Interproximal contacts			
1: Poor	2 (22.2)	4 (44.4)	0.69
2: Unsatisfactory	2 (22.2)	1 (11.1)	
3: Good	5 (55.6)	4 (44.4)	
4: Excellent	0 (0.0)	0 (0.0)	
WES			
Tooth form			
0	0 (0.00)	1 (11.1)	0.99
1	2 (22.2)	2 (22.2)	
2	7 (77.8)	6 (66.7)	
Tooth volume/outline			
0	0 (0.0)	0 (0.0)	0.33
1	2 (22.2)	5 (55.6)	
2	7 (77.8)	4 (44.4)	
Color hue/value			
0	1 (11.1)	0 (0.0)	0.13
1	7 (77.8)	4 (44.4)	
2	1 (11.1)	5 (55.6)	
Surface texture			
0	2 (22.2)	0 (0.0)	0.05
1	6 (66.7)	3 (33.3)	
2	1 (11.1)	6 (66.7)	
Translucency			
0	0 (0.0)	1 (11.1)	0.99
1	8 (88.9)	7 (77.8)	
2	1 (11.1)	1 (11.1)	
Total	7.00	6.56	0.45
PES			
Mesial papilla			
0	1 (11.1)	3 (33.3)	0.29
1	8 (88.9)	5 (55.6)	
2	0 (0.0)	1 (11.1)	
Distal papilla			
0	3 (33.3)	3 (33.3)	1.00
1	5 (55.6)	6 (66.7)	
2	1 (11.1)	0 (0.0)	
Soft tissue level			
0	0 (0.0)	0 (0.0)	0.47
1	2 (22.2)	0 (0.0)	
2	7 (77.8)	9 (100.0)	
Soft tissue contour			
0	0 (0.0)	0 (0.0)	1.00
1	3 (33.3)	4 (44.4)	
2	6 (66.7)	5 (55.6)	
Alveolar process deficiency			
0	0 (0.0)	0 (0.0)	1.00
1	1 (11.1)	1 (11.1)	
2	8 (88.9)	8 (88.9)	
Soft tissue colour			
0	0 (0.0)	0 (0.0)	0.029
1	0 (0.0)	5 (55.6)	
2	9 (100.0)	4 (44.4)	
Soft tissue texture			
0	0 (0.0)	0 (0.0)	0.029
1	2 (22.2)	5 (55.6)	
2	7 (77.8)	4 (44.4)	
Total	11.11	9.78	0.057

### Patient-reported outcome measures (PROMs)

The satisfaction scores displayed in Table 4 were similar between the groups, except for the comfort during the polyether impression (control group) vs. digital impression (test group); the digital impression was found to be significantly more comfortable compared to the conventional one (*p* = 0.014).

**Table 4:** PROMs (patient-reported outcome measures)

	Test (*N* = 9)	Control (*N* = 9)	*p*-value
1. Feeling about the provisional restoration	8.44	7.56	0.081
2. Natural aspect of the teeth	8.00	7.89	0.92
3. Impression discomfort	1.1	5.00	0.014
4. Treatment compliance	10.00	9.56	0.085
5. Esthetic satisfaction	9.00	8.78	0.75

Nevertheless, the esthetic results from the patient point of view (esthetic VAS) showed no statistically significant differences between the control group and the test group (*p* = 0.75). In the control group, the comparison between the VAS (8.8 ± 1.1) and the WES (7.0 ± 1.2) showed a significant difference between the perception of the esthetics of the provisional restoration for the dentist and the patient (*p* = 0.0035). A similar observation was made in the test group, VAS (9.0 ± 1.7), WES (6.6 ± 1.2), (*p* = 0.0002). The details of the results are shown in Table 4.

## Discussion

The present randomized controlled trial compared a fully digital vs. a conventional workflow for immediate restoration of a single implant in the esthetic zone using a one-abutment one-time protocol. Based on the surgical planning, a higher surgical accuracy was found with guided surgery compared to the free-handed surgery; however, the immediate restoration procedure was successful for all patients from both group. The clinical fit of the provisional restorations (occlusion and interproximal contacts), as well as the WES were comparable in both groups. Moreover, PROMs revealed to be similar between the groups, except for less discomfort found with digital impressions in the test group compared to the conventional polyether impressions used in the control group.

### Surgical outcomes

The accuracy of the implant position is usually evaluated by comparing the deviation of the entry point and the apex position, as well as the angulation of the implant, with the implant planning [28]. In this study, a statistically significant difference was found between s-CAIS and free-handed surgery for the angulation (2.41° in the s-CAIS group vs. 6.26° in the free-handed group) and for the apical deviation (1.36 in the s-CAIS group vs. 2.42 in the free-handed group).

Several reviews and meta-analysis explored the accuracy of implant placement [23, 26, 34]. Most of these reviews included bone, mucosa and tooth supported guides in complete and partially edentulous patients, in vivo and in vitro studies, and different planification softwares, which makes the comparison with the present study rather difficult. However, in general, higher accuracy was found in partially edentulous patients with tooth supported surgical guides compared to fully edentulous patients [28, 34].

When considering guided vs. free-handed surgery for single tooth replacement, the results of the present study are similar to those of Smitkarn et al. [35], emphasizing a higher accuracy with the s-CAIS surgical guide compared to the free-hand one. They found a significant difference between the groups for angulation, deviation at the entry point and at the apex, while in the present study, the differences between the groups were mainly found for angulation and at the apex. Another split mouth design study using a different software and implant system concluded that computer generated surgical guides were generally closer to the planned position although they could find a significant difference only for coronal horizontal distance [16]. However, the authors reported the fit of the CAD–CAM guides as a limitation, as a relining procedure was often necessary to enhance stability prior to the surgery. In the present study, only one s-CAIS guide did not display an immediate fit and had to be adjusted, compromising its stability. As a result, this case displayed the highest deviation at the entry point (1.13 mm). As mentioned by many authors, one of the crucial factors for precision is the stability of the template position during implant placement [24, 28, 36–38].

Another complication occurred with the s-CAIS guide in one patient: 2 mm over drilling was observed during the surgery. It seems that an unidentified issue occurred in the digital workflow, and it is important to note that the digital workflow is prone to errors that might happen throughout the multiple steps and have a cumulative effect. Fortunately, in this specific case, no anatomical structures were injured. This incident may reinforce the idea of keeping a safety zone of at least 2 mm to avoid critical anatomical structures’ injuries as suggested by Casetta et al. [39].

Several designs of drilling systems are available for s-CAIS, including sleeve-in-sleeve system, sleeve-in-sleeve with self-locking, the mounted sleeve-on-drill, the integrated sleeve-on-drill, and the integrated sleeve-on-drill without a metal sleeve. The sleeve-in-sleeve system, used in the present study, was shown to lead to significantly less angular deviation compared to other systems listed above [40]. Regarding the IOS, the accuracy of IOS for single implant was widely demonstrated [41, 42] and should, therefore, be considered as a valid research tool complying with (ALADA) principles for patient radiation safety [43–46].

Finally, it must be emphasized that the concept of implant placement accuracy should be interpreted with caution. Indeed, there is not a single optimal implant position and the surgical planning may not be the absolute gold standard. Moreover, in some cases, certain deviations might be more important than others, if, for instance, important anatomic structures might be endangered or limited bone volume is present, while, in some other cases, a statistically more significant deviation relative to planning might still result in a satisfactory prosthetic outcome. In the present study, all the implant positions provided a good setting for a restorative phase, despite the deviations between the planification and the final position of the implant. A relevant impact of surgical experience on the accuracy of implant placement is to be expected, especially for free-handed surgery.

### Provisional restoration outcomes and loading procedure

In the present study, the clinical fit of the provisional restorations (occlusion and interproximal contacts) was similar in the two groups. Although the possibility to perform a single unit provisional crown, and therefore, an immediate loading based on the surgical planning was already described in case reports [47, 48], the present trial validates the concept for the first time when compared with a conventional approach. Although these results are promising, a longer follow up period is necessary to exclude some succeeding, potentially undesired events that could be related to this treatment.

This new workflow allows a soft-tissue-friendly one-abutment one-time immediate restoration with a custom-made abutment respecting the optimal cervical profile and limiting adverse events, such as cement excess.

The esthetic outcome of an implant restoration is also a critical parameter for implant success, especially since patient expectations tend to increase nowadays. Although no significant difference was found for the WES score between the two groups, a trend for a better surface texture was found in the control group in which a stratified PMMA was performed. The surface texture discrepancies observed with the CAD–CAM PMMA provisional are most likely related to the manufacturing process of crowns using monochromatic blocks. However, the esthetic discrepancies found in the present study are very limited and did not compromise patient satisfaction.

### Patient-reported outcome measures (PROMs)

Nowadays, PROMs have become an important parameter in evaluating treatment success. In the present study, all 18 patients were satisfied with the esthetic aspect of their crown. However, there are few controlled studies about patient-centered outcomes for single implant treatment and there is a lack of recommendation on the assessment method [49]. The satisfaction questionnaire used in the present study might be useful in the absence of other tools. The present results demonstrate that both protocols are highly accepted by patients. The only difference between the groups was observed for the impression (IOS vs. polyether impression); the patients reported more discomfort with the conventional impression. Overall, the present results are in accordance with the literature, highlighting that patients preferred the digital impressions over the conventional methods [50, 51].

The present findings must be interpreted cautiously, taking into consideration a relatively short follow up period, but also because the present outcomes could be related to the growing interest of people for new digital technologies and the increase of their use in everyday life (smartphones, tablets, etc.). Therefore, as already suggested by some authors, the perception of new technologies may have influenced the outcome [52]. Furthermore, the present RCT considered both healed and fresh extraction sites, and only anterior region (from central incisors to premolars), which might have impacted the precision of the surgical preparation and implant insertion, and hence the overall results as well. Further clinical aspects of the fully digital approach, such as stability of peri-implant soft tissues, should be assessed following a longer follow up period. In addition, the time consumption (overall treatment time needed for planning, surgery, impression, prosthetic delivery as well as prosthetic adjustments) and cost‐effectiveness of the fully digital approach need to be further studied, as both are relevant factors to be taken into consideration in clinical decision-making.

## Conclusion

Within the limitations of the present study, the following conclusions can be drawn:

- Both workflows (conventional and fully digital) allowed implant placement and immediate or early restoration of the implant using a one-abutment one-time procedure;
- The fully digital group was associated with more accurate implant placement compared to conventional implant placement;
- Both workflows resulted in acceptable and comparable clinical and esthetic outcomes (contact points, occlusion, and WES);
- Higher patient satisfaction (in terms of comfort) was achieved using the IOS over the conventional polyether impression.

## Data Availability

Not applicable.

## Abbreviations

AD: Angular deviation
AAD: Apical apical deviation
ADD: Apical distal deviation
AVD: Apical vestibular deviation
CAD: Coronal apical deviation
CAD–CAM: Computer-aided design and computer-aided manufacturing
CBCT: Cone-beam computed tomography
CDD: Coronal distal deviation
CGD: Coronal global deviation
CVD: Coronal vestibular deviation
GAD: Global apical deviation
IOS: Intra oral scan
PES: Pink esthetic score
PMMA: Polymethyl methacrylate
PROMs: Patient-reported outcome measures
s-CAIS: Static computer‐assisted implant surgery
VAS: Visual analogue scale
WES: White esthetic score
